# Supervised binary classification methods for strawberry ripeness discrimination from bioimpedance data

**DOI:** 10.1038/s41598-021-90471-5

**Published:** 2021-05-27

**Authors:** Pietro Ibba, Christian Tronstad, Roberto Moscetti, Tanja Mimmo, Giuseppe Cantarella, Luisa Petti, Ørjan G. Martinsen, Stefano Cesco, Paolo Lugli

**Affiliations:** 1 Faculty of Science and Technology, Free University of Bolzano-Bozen, 39100 Bolzano-Bozen, Italy; 2 Department of Clinical and Biomedical Engineering, Oslo University Hospital, Oslo, 0315 Norway; 3 Department for Innovation in Biological, Agro-Food and Forest Systems, University of Tuscia, 01100 Viterbo, Italy; 4 Competence Centre of Plant Health, Free University of Bolzano-Bozen, Piazza Universitá 1, 39100 Bolzano-Bozen, Italy

**Keywords:** Plant sciences, Techniques and instrumentation, Electrical and electronic engineering

## Abstract

Strawberry is one of the most popular fruits in the market. To meet the demanding consumer and market quality standards, there is a strong need for an on-site, accurate and reliable grading system during the whole harvesting process. In this work, a total of 923 strawberry fruit were measured directly on-plant at different ripening stages by means of bioimpedance data, collected at frequencies between 20 Hz and 300 kHz. The fruit batch was then splitted in 2 classes (i.e. ripe and unripe) based on surface color data. Starting from these data, six of the most commonly used supervised machine learning classification techniques, i.e. Logistic Regression (LR), Binary Decision Trees (DT), Naive Bayes Classifiers (NBC), K-Nearest Neighbors (KNN), Support Vector Machine (SVM) and Multi-Layer Perceptron Networks (MLP), were employed, optimized, tested and compared in view of their performance in predicting the strawberry fruit ripening stage. Such models were trained to develop a complete feature selection and optimization pipeline, not yet available for bioimpedance data analysis of fruit. The classification results highlighted that, among all the tested methods, MLP networks had the best performances on the test set, with 0.72, 0.82 and 0.73 for the F$_1$, F$_{0.5}$ and F$_2$-score, respectively, and improved the training results, showing good generalization capability, adapting well to new, previously unseen data. Consequently, the MLP models, trained with bioimpedance data, are a promising alternative for real-time estimation of strawberry ripeness directly on-field, which could be a potential application technique for evaluating the harvesting time management for farmers and producers.

## Introduction

Strawberry is a widely popular and high valued non-climateric fruit cultivated in many areas, from Europe to Asia and Americas and also extensively traded all over the world^1^. While the appearance of the strawberries (i.e. color, shape, size, and presence of defects) plays the most important role in the determination of its quality, also its flavor and nutritional composition are important^2^. Most of the aforementioned factors depend on the physiochemical changes occurring during the on-plant ripening of strawberry fruit, which must be harvested at its optimal ripeness stage (commonly when > 80% of the fruit surface shows a deep red color^3^) in order to achieve its maximum quality^4^. Usually, strawberry quality is manually and visually graded by farm-labors as well as analytically by applying destructive approaches in specialized labs. However, such type of classification is not completely reliable, as it cannot guarantee consistent grading due to human judgement subjectivity and errors, in addition to requiring considerable time and effort^5^. For this reason, the fruit industry has recently experienced an increased demand for quality monitoring techniques able to rapidly, precisely, and non-destructively classify such fruit maturity stage, allowing thus to reduce wastes and increase market value. Among such techniques, the most widely employed methods to evaluate strawberry fruit quality include image analysis^6^, hyperspectral and multispectral imaging^7,8^, near infrared spectroscopy^9^ and more rarely electronic nose^10^ and laser induced fluorescence^11^.

In this context, electrical impedance spectroscopy (EIS), also called bioimpedance when applied to biological tissues, represents an especially interesting alternative, due to its simplicity, non-invasiveness, and cost-effectiveness. This method, already used in many different contexts, from food product screening^12^ to solid materials properties characterization^13^ and human body analysis^14^, is based on the physico-chemical property of a target to oppose to the flow of current induced by an external AC voltage applied at multiple frequencies^15^. The resulting bioimpedance output depends on many factors, such as the geometry of the object, the electrical properties of the material and the chemical processes that take place inside it^16^. In the context of EIS-based classification methods, the major challenges are represented by the intrinsic nature of the bioimpedance data, commonly acquired as frequency spectrum. Indeed, such data are represented by a combination of different electrical parameters, which have highly correlated frequency points and often have a non-linear association with the physico-chemical behavior of the product. Usually, as the relevant information is often contained in only a small portion of the frequency spectrum, bioimpedance data are reduced into a small set of variables accounting for most of the information in the measurements^15^. To achieve this, such data are typically fitted in an electrical equivalent circuit, such as the Cole model^17^, reducing the measurement into few uncorrelated parameters, easier to handle statistically but not always compatible with the data^18^. An alternative solution to the above-mentioned issues, employed mostly in a biomedical context, is represented by the use of machine learning based classification methods. Within this framework, the most widely used and effective discrimination techniques are represented by LR^19^, artificial neural networks (ANN)^20^, DT^21^, SVM^22^, NBC^23^ and KNN^24^.

In the specific case of strawberry fruit, the works available in literature on bioimpedance concern (i) the detection of fungal diseases^25^, (ii) the evaluation of the fruit ripeness^26^, and (iii) the post-harvest aging evolution^27^. Nevertheless, all these works lack of well-established machine learning methods predicting the fruit ripening stage starting from bioimpedance data. The most relevant works in this specific field describe the application of support-vector machine algorithms to the classification of avocado ripening degree^28^ and the detection of freeze damage in lemons using artificial neutral networks and principal component analysis^29^. Both papers achieved a good accuracy in the classification task, obtaining a 90% (SVM) and 100% (ANN) precision, training the models with a total 100 and 180 samples, respectively. Such works, despite being a good starting point for the use of a combined bioimpedance and machine learning approach for the evaluation of fruit quality, lack in the amount of considered data to develop the models and most importantly do not provide a detailed data analysis pipeline, which is strongly needed as a reference in the development of similar works.

Motivated by these limitations, in this work we investigate the use of the 6 most common machine learning binary classification methods, described briefly in the methods section, as well as their application in the discrimination of strawberry ripeness from bioimpedance on-plant measurements. Specifically, we first describe the data acquisition and transformation process, followed by a first manual and a second automatic feature selection. Secondly, we describe the algorithms optimization and the selection of the best classification algorithms, including the importance of the selected features for each different method. Finally we describe and discuss the results of the classification on the external, and unseen, dataset. Such models are developed to be coupled with already developed portable instruments^30^ and employed for the on-field strawberry fruit quality assessment to facilitate and optimize the harvesting process.

## Methods

### Plant material

Commercially available strawberry plant material ($Fragaria \times ananassa$ cv. Elsanta) was kindly provided by Dr. Gianluca Savini from Cooperativa Sant’Orsola, Pergine Valsugana (TN), Italy. A total of 80 strawberry frigo-plants were planted using a commercially available soil substrate in individual pots and then cultivated in a growth chamber under controlled conditions (day 14 h, 24 °C, 70 % relative humidity, 250 µmol photons m$^{-2}$ s$^{-1}$; night 10 h, 19 °C, 70 % relative humidity). Plants were grown for 30 days and were maintained at approx. 60 % water holding capacity, by watering them twice a week with tap water.

### Experimental setup

**Figure 1:**
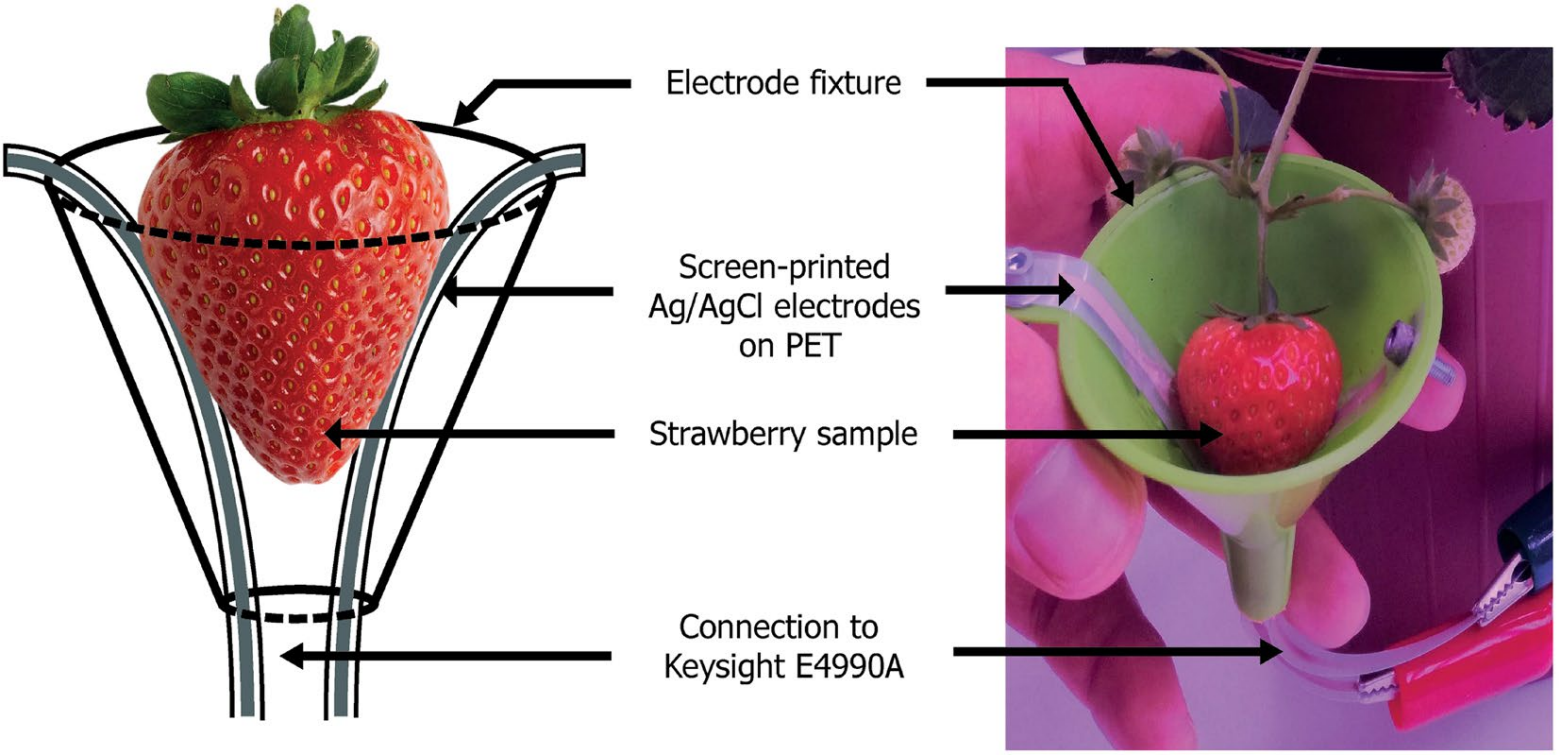
Measurement setup for the strawberry on-plant bioimpedance data acquisition, including the schematic (left) and the real application (right).

The strawberry quality was monitored during the experiment by acquiring fruit size, skin color and bioimpedance data. The size was acquired by means of a caliper by measuring the fruit maximum diameter. Strawberry skin color was evaluated determining the color parameters in the CIELab color space using a portable tristimulus colorimeter (Chroma Meter CR-400, Konica Minolta Corp., Osaka, Japan). Finally, the strawberry bioimpedance data were acquired using an E4990A bench-top impedance analyzer (Keysight Technologies, Santa Rosa, CA, USA) in the 20 Hz–300 kHz frequency range, over 300 logarithmically spaced frequency points, in a two-electrodes configuration. The electrical contact with the fruit was established by means of custom-made screen-printed Ag/AgCl electrodes together with contact gel (FIAB G005 high condictivity ECG/EEG/EMG gel). To compensate the electrode effect on the measurement, especially at high and low frequencies and considering the use of custom-made electrodes, the system was calibrated by means of a open/short/load (load = 15 k$\Omega$ resistor) calibration procedure. As shown in Fig. 1, the electrodes were embedded on a cone shaped fixture, which allowed to easily contact the fruit directly on-plant and from the bottom, thus achieving the electrical contact solely by means of the fruit weight. Furthermore, the electrolyte gel used to improve the electrical contact resulted not to harm the fruit and to be easily washable, leaving the fruit intact after each data acquisition. Hence, no fruit were wasted during the data collection campaign, resulting in a total of 923 measured strawberries: each fruit size, skin color and bioimpedance was monitored daily, directly on plant, from the fruit onset to its harvest at full maturity stage, i.e. when > 80% of the fruits surface showed a deep red color^3^.

### Sample preparation

The strawberry fruits were split in two classes, namely ripe and unripe, based on the commonly used a* coordinate, which ranges from negative to positive values for green and red tones, respectively^31^. The discrimination threshold was set to a* = 35, selected to be the optimal value according to Nunes et al.^32^ and Kim et al.^33^. The total 923 sampled strawberries were divided in two sets of data of 684 and 239 samples, to be respectively used in the training and testing of the classification algorithms. Each set was then divided in the two ripening classes, used as labels in the binary classification procedure training and testing. The training set resulted to be composed of 534 unripe and 150 ripe samples, while the testing set was composed of 128 and 111 unripe and ripe samples, respectively.

### Bioimpedance data pretreatment and manual feature selection

**Figure 2:**
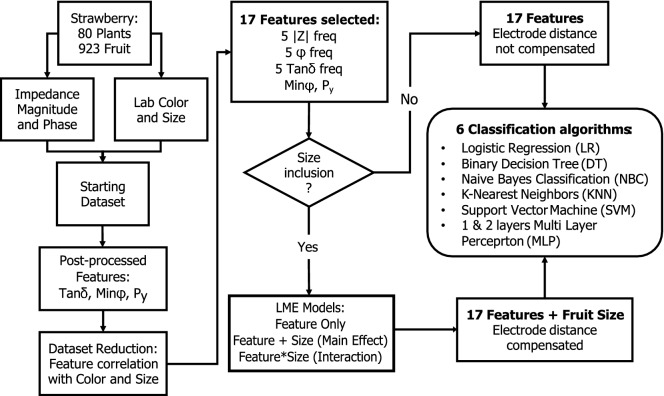
Overview of the methodology used for the investigation of the most relevant features of the bioimpedance dataset, also including the fruit size as additional feature to compensate the electrode distancing, to be used to train the classification algorithms.

Prior to the model development, additional features such as (i) the minimum phase point of each spectrum, (ii) the P$_y$^34^ and (iii) the tan $\delta$^35^ parameters were extracted from the raw magnitude and phase angle spectra (Fig. 2). In this context, the commonly used fitting of the data to equivalent circuit models was not employed, due to two main drawbacks: (i) the acquired bioimpedance curves had a poor fitting quality with two reference circuits, i.e. the Cole model^17^ and the model developed by Ibba et al.^36^, making unreliable the extraction of the circuit parameters; (ii) the P$_y$ parameter has a relationship with both the high and low frequency resistors as well as the constant phase element (CPE) values of the Cole model, which would supply mostly redundant information to be used in the model optimization. In particular, the former fitting quality issue resulted to be associated with the measurement frequency range (20 Hz–300 kHz), which was chosen based on previous studies on different fruit. In fact, the Cole model R$_\infty$ value, related to the high frequency resistance, is commonly estimated at frequencies higher than our upper limit (300 kHz), where the magnitude curve is completely flattened. This caused the inaccurate estimation of such value, inevitably leading to wrongfully estimate the other circuit parameters, thus providing unreliable values which could not be used in the discrimination model development. Consequently, the fitting of the data with other models implementing the R$_\infty$ component, or introducing a too high degree of complexity was completely excluded. This was also based on the use of the above-mentioned P$_y$ parameter and on the fact that a significant reduction of the data was already achieved with other methods.

**Figure 3:**
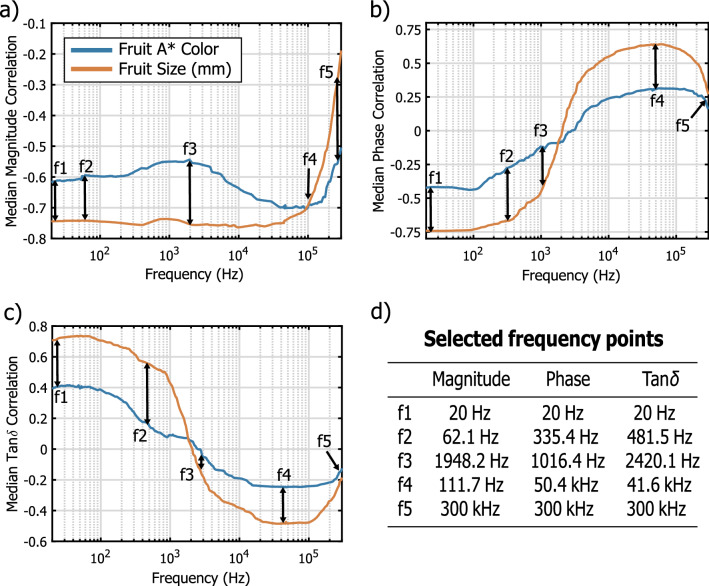
Median correlation of the fruit color (blue line) and size (red line) with the bioimpedance magnitude (**a**), phase (**b**) and tan $\delta$ (**c**) parameters, together with the 5 selected frequency points for each parameter (**d**).

Afterwards, the fruit bioimpedance magnitude, phase and tan $\delta$ spectra values, for each frequency point, were tested in terms of their correlation with both fruit size and color, to find and select frequency points correlating more with the fruit color change and less with the fruit size. In fact, while the former precisely relates to the fruit ripening stage, the latter does not have a direct relationship with it. Furthermore, the electrodes were contacting each fruit at a different distance due their different size. Hence, the selection of frequencies that poorly correlated with the size, may help in the exclusion of this source of variability in the classification models building. From the correlation results, shown in Fig. 3, it is possible to notice that the bioimpedance magnitude had better correlation with fruit size than with redness, except for the high frequencies (Fig. 3a). Similar results were observed for both phase and tan $\delta$ parameters (Fig. 3b,c, respectively), which however, as expected, showed opposite trends due to their relationship with the phase angle ($\delta = 90^{\circ }- \phi$)^15^. Due to this and to the high autocorrelation of neighboring frequencies in the impedance spectrum, 5 frequency points for each parameter were selected, at the most interesting correlation behavior (e.g. intersection and largest distance of the two median correlation curves). The selected frequency points are indicated in Fig. 3d. After the above-mentioned steps, the dataset resulted to be composed of a total of 17 features, namely 5 frequencies points for the impedance magnitude, phase and tan$\delta$ each, plus both P$_y$ and minimum phase values.

#### Size inclusion in features using LME models

As discussed above, the strawberry fruit size might have had a significant effect on the measured impedance spectrum. For this reason, the Linear Mixed Effect model (LME) was used as an easily interpretable tool to understand the relationships among variables and investigate the inclusion of the fruit size as additional feature, to improve the classification model performance. The test was carried out by considering the fruit color (i.e. redness a*) as the response variable “y”, the selected 17 feature as the fixed effect “X” and the fruit size as the random effect “Z”. Subsequently, each of the 17 features ($X_i$) was used to explain the changes in fruit color (y) (i) with a “feature only” model ($y\,\sim \,X_i$), considering only the effect of the bioimpedance feature ($X_i$) on the color change, (ii) with a “main effects” (or additive) model (Feature + Size, i.e. $y\,\sim \,X_i\,+\,Z$), considering the effects of both $X_i$ and Z on the color change and (iii) with an interaction model (Feature × Size, i.e. $y\,\sim \,X_i\,^{*}\,Z$), considering both the interaction and the main effect of the two features $X_i$ and Z. Afterwards, the differences among the 3 cases were evaluated using the likelihood ratio test^37^, which was used to compare the goodness of fit of each pair of models based on the ratio of their likelihoods (Table 1). As result, a *p*-value $\le 0.05$ indicated that the more complex model was significantly better than the simpler one in the evaluation of the response variable, i.e. fruit redness (y).

**Table 1:** Significance of the Linear Mixed Effect model quality improvement for each of the 17 selected features with the addition of the size and the interaction between the size and the feature.

Compared models	|Z| (f1)	|Z| (f2)	|Z| (f3)	|Z| (f4)	|Z| (f5)	$\phi$ (f1)	$\phi$ (f2)	$\phi$ (f2)	$\phi$ (f4)
Feature only vs. main effect	$\le \,0.05$	$\le \,0.05$	$\le \,0.05$	$\le \,0.05$	$\le \,0.05$	$\le \,0.05$	$\le \,0.05$	$\le \,0.05$	$\le \,0.05$
Main effect vs. interaction	0.55	0.97	0.39	$\le \,0.05$	$\le \,0.05$	0.86	0.64	0.56	0.08
Compared models	$\phi$ (f5)	tan $\delta$ (f1)	tan $\delta$ (f2)	tan $\delta$ (f3)	tan $\delta$ (f4)	tan $\delta$ (f5)	P$_y$	Min $\phi$	–
Feature only vs. main effect	$\le \,0.05$	$\le \,0.05$	$\le \,0.05$	$\le \,0.05$	$\le \,0.05$	$\le \,0.05$	$\le \,0.05$	$\le \,0.05$	–
Main effect vs. interaction	0.84	$\le \,0.05$	0.39	0.09	1	0.79	0.15	1	–

*|Z|* impedance magnitude, $\phi$ impedance phase, – no feature.

Table 1 shows the results of the LME tests. In general, the nested mixed models were significantly better than fixed models, while the crossed mixed models were effective for just 3 fixed effects. In short, the LME results suggest that the classification would benefit from the inclusion of size as a “correction” feature, in addition to the bioimpedance-derived ones, as it has most likely had an impact on the measurement, and thus results useful to mitigate the effect of the different electrode placement on the bioimpedance data acquired from different fruit.

### Fruit ripeness classification

**Figure 4:**
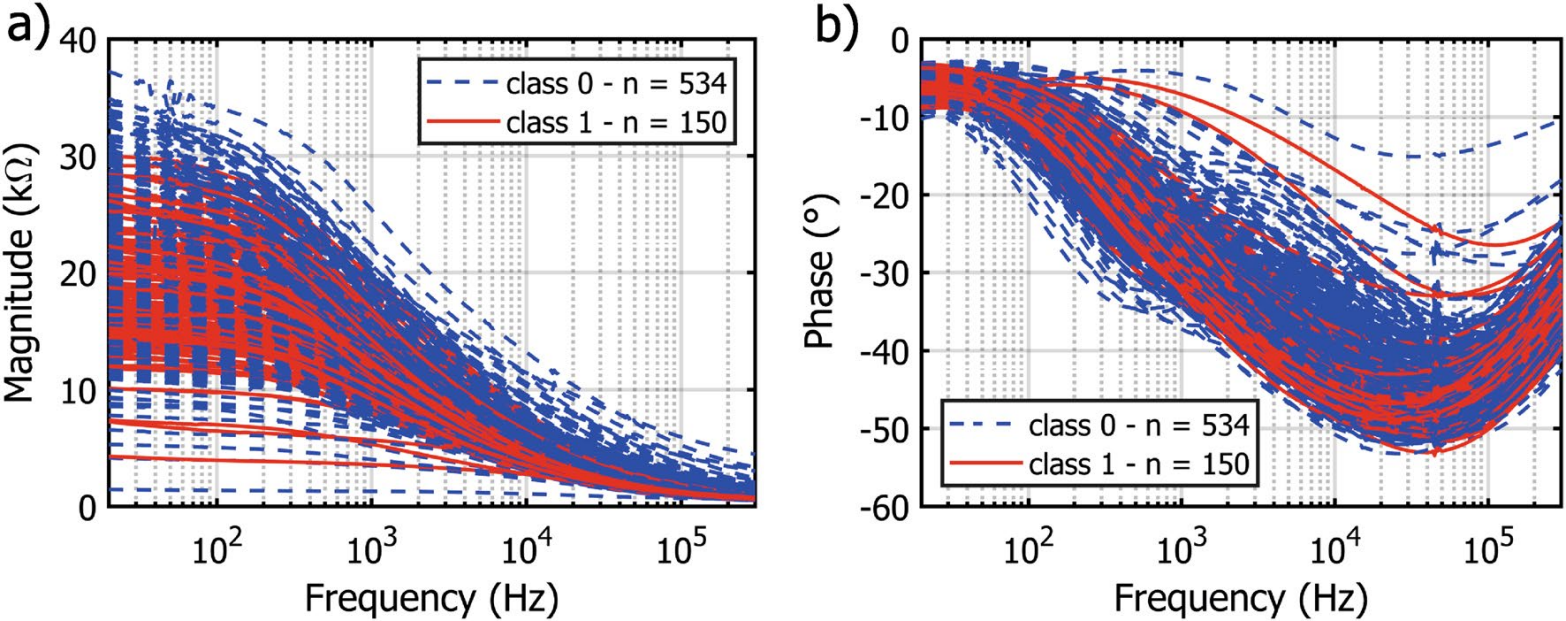
Overview of the training set of bioimpedance magnitude (**a**) and phase (**b**) spectra for both classes of ripeness, ripe (class 1, red line) and unripe (class 0, blue line).

After evaluating the most relevant features to be used in the classification, the distribution of the two classes (ripe and unripe) for the training set was evaluated, to optimize the discrimination procedure. The main problems to be considered in the development of the procedure, also visible from the bioimpedance magnitude and phase spectra shown in Fig. 4, were represented by the high degree of overlap and by the skewed distribution of the two considered classes.

The latter was solved by considering the prior probability (i.e. the relative frequency with which observations from that class occur in a population) of both classes as uniform, attenuating the class imbalance effect on the classification. The former was tackled by testing different type of machine learning classification algorithms, introduced hereafter with their main characteristics, to evaluate the one with better performance in discriminating the fruit ripening. *Logistic Regression (LR)*^38^ is a simple form of non-linear regression which uses a logistic function to model a binary dependent variable. It assumes a linear relationship between the log-odds (the logarithmic of the odds) of the classes and one or more independent variables, or predictors. *Decision trees (DT)*^39^ are a non-parametric supervised machine learning method which creates a model that predicts the class of a target variable by learning simple decision rules inferred from the data features. The main advantage is that the generated models can be easily interpreted. On the other hand, the DT model might suffer from lack of generalization (or overfitting) and small data perturbations might result in completely different trees being generated. A *Naive Bayesian Classifier (NBC)*^40^ is based on the assumption that all features are conditionally independent given the class variable and that each distribution can be evaluated independently as a one dimensional distribution. This method, while being less sophisticated than others, has proven to be fast and effective, producing good, simple models requiring little training. *K-Nearest Neighbors (KNN)* classification^41^ is a method which assumes that samples belonging to the same class are similar to each other. It calculates the distance between those points, assigning a class membership based on the most common class assigned to its *k* nearest neighbor. *Support Vector Machine (SVM*)^42^ separates data by drawing hyper-planes in the N-dimensional feature space (N $=$ the number of features) and finds the one which provides the maximum distance between data points of both classes, maximizing the class separation. A *Multi Layer Perceptron (MLP)*^43^ is the simplest feed-forward neural network. It consists of at least three layers of nodes: an input layer with number of nodes equal to that of input features, a hidden layer and an output layer with number of nodes equal to that of output classes. Except for the input nodes, each node uses a nonlinear activation function. Such networks have the capability of learning non-linear models.

Before starting the training procedure, the features used in the classification were normalized by calculating the vectorwise z-score of each feature with center 0 and standard deviation 1, to exclude the influence of different unit of measurement and absolute values, thus enhancing the reliability and the comparability of the trained models results^44^. The F-score was used as the figure of merit to evaluate the optimization, training, and testing performance of each algorithm and to compare the different applied methods between each other. This metric was selected as the most appropriate for this study case due to the skewed training classes division, for which the most commonly used classification accuracy metric would not have been completely representative. The F-score is defined as the harmonic mean of precision (P), also defined as the accuracy of the positive prediction, and recall (R, also called sensitivity), which is the ratio of the positive instances correctly detected by the classifier. In particular, it was used the F$_\beta$-score, a derivation of the F-score, defined as follows^45^:

1 $$\begin{aligned} F_\beta = \frac{(\beta ^2 + 1)PR}{\beta ^2P + R} \; \; \; (0 \le \beta \le +\,\infty ), \end{aligned}$$

where $\beta$ is a parameter that controls the balance between P and R. When $\beta = 1$, F is the equivalent of the harmonic mean of P and R, while if $\beta > 1$, F gives more importance to recall (R) and if $\beta < 1$, it gives more weight to precision (P)^46^. In this paper, the model performances were evaluated in terms of F$_\beta$-scores with $\beta$ values of 1, 0.5 and 2, to obtain and select the best models for a balanced, precision-oriented and recall-oriented classification, respectively.

### Optimization of classification algorithms and sequential feature selection

**Figure 5:**
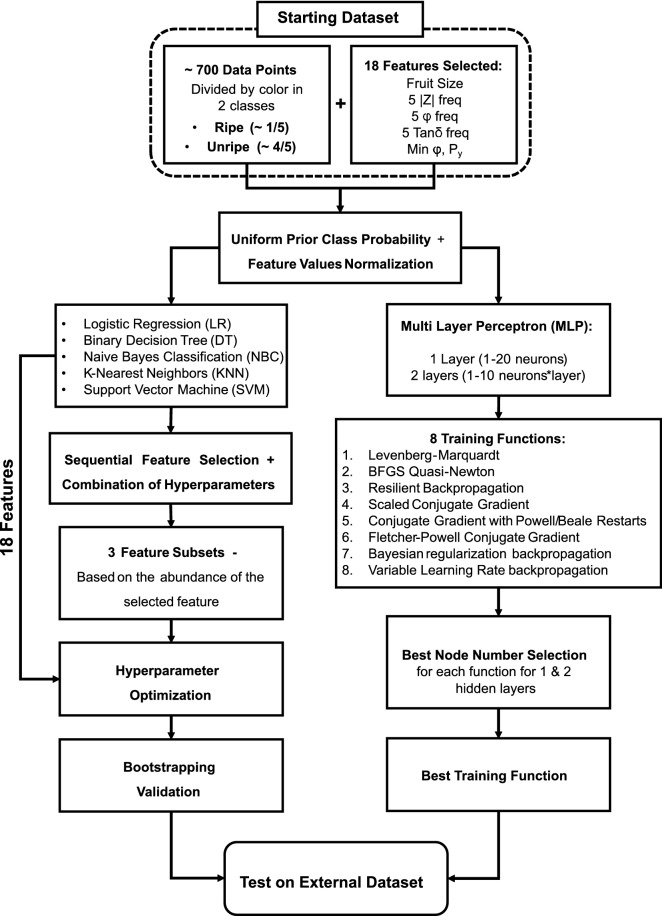
Overview of the training methodology used for the algorithm optimization and sequential feature selection. The left side represents the methodology used for LR, DT, NBC, KNN and SVM. The right side the one used for MLPs with 1 and 2 layers of hidden nodes.

The six classification algorithms were developed, as schematized in Fig. 5, using two different approaches. In fact, while MLPs were optimized using the entirety of the 18 selected features, for LoR, DT, NBC, KNN and SVM an automatized feature selection was adopted. This is because the majority of the latter models benefit from the use of a lower number of features, in terms of classification speed and interpretability of the impact of each variable on the classification outcome.

The optimization of the LoR, DT, NBC, KNN and SVM models consisted in an automatic backward feature selection combined with a grid search of combination of hyperparameters. In practice, starting from all the 18 features, the feature selection function sequentially removed every feature from the dataset to check if it produced any improvement in the classification task, which was evaluated in terms of F$_\beta$-score with a fixed tenfold cross-validation. The feature selection procedure was carried out for all the possible features and hyperparameters combinations and the relative frequency with which a feature was selected was used to define its importance in the model. Results allowed the creation of 3 different feature subsets for each classification algorithm, based on the selection of 3 numerosity thresholds, set by visual evaluation to select approximately 2/3, 2/4 and 1/3 of the 18 starting features. Consequently, 20 subsets (including the one with all the 18 features) were obtained for each model. Afterwards, each subset underwent a final automatic hyperparameter optimization through the maximization of the F_1_, F_0.5_ and F_2_ scores on the training dataset. Each algorithm performance was then subjected to a 10,000-rounds bootstrap validation using the training dataset. Each optimized model was then tested on the external dataset.

The MLP models considered in this work were composed of 1 and 2 hidden layers of nodes, to obtain a simple network to compare the results of the previously optimized methods. The optimization of such networks consisted in the training of 8 different training functions (see list in Fig. 5) with an increasing number of nodes, up to a maximum of 20, distributed in 1 or 2 hidden layers. This procedure was carried out by splitting the the original training set into a reduced training set (60% of samples), validation set (20% of samples) and internal test set (20% of samples), to develop a prediction model to be tested on the external test set. The performance of each model was evaluated in terms of F$_\beta$-scores for the prediction of the internal test dataset portion, to select the optimum number of neurons for each function. Afterwards, the optimized functions were further tested to evaluate which had the best performances on a second 60:20:20 random division of the reduced training set, and the best training function was selected, for both the 1 and 2 layers network, to be applied for the classification on the external dataset.

## Results and discussion

### Feature reduction and selection

**Figure 6:**
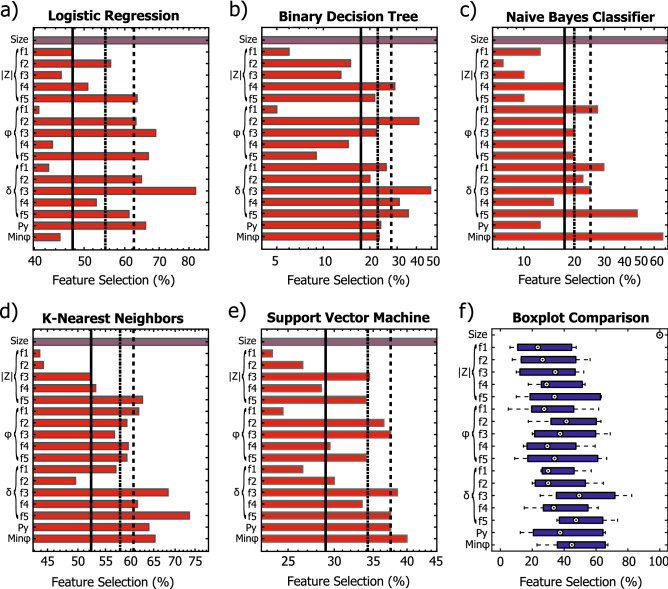
Sequential feature selection of the starting 18 parameters, together with the 3 selected numerosity thresholds (low solid line, medium dash dotted line, high dashed line) for each model (**a**–**e**). Boxplot comparison (**f**, blue filled square) of the feature selection among the models. As decided form previous considerations, the fruit size feature (violet filled square) is always selected, the 17 other features (ref filled sqaure) are subject of the feature selection.

Figure 6a–e depicts the sequential feature selection results for the *LR, DT, NBC, KNN and SVM* algorithms, together with the applied numerosity thresholds and with the boxplot comparison (Fig. 6f) for each feature for all the classification methods.

The different thresholds were set to test the models in different numerosity conditions and not to exclude specific features. However, this method resulted to be also useful to understand the importance of each bioimpedance-derived parameter in the development of the classification methods. At this stage, it is important to underline that in poorly performing models (*LR, DT and NBC*, Fig. 6a–c) the selected features significance provides less information, as a poor fit with the training data might be associated with an arbitrary feature selection.

Among the features related to the impedance magnitude, namely the 5 selected frequencies and the P$_y$, it is noticeable (Fig. 6a,b,d,e) how, for this specific binary classification task, the low frequency points (f1 and f2) are the least selected, while the medium to high frequency points (from f3 to f5) and the P$_y$ parameter resulted to be more relevant in the class discrimination. The latter, being typically less dependent on the electrode positioning compared to the magnitude at specific frequency points, might be considered as a valid alternative to reduce (or even completely exclude) the use of such features, strongly affected by the electrode positioning, hence reducing the classification error introduced by this issue. The phase-related features, the minimum phase point and the 5 selected frequencies for the phase angle ($\phi$) and tan $\delta$, are also commonly considered to be less affected by the measurement setup compared to the magnitude-related data^47^. In the specific case of the *KNN*, one of the best performing and stable method (Fig. 6d), the tan $\delta$ at medium to high frequency (from f3 to f5) and the minimum phase point resulted to have a high impact in the model development, while the overall phase angle features ($\phi$ from f1 to f5) resulted to have a medium importance. The same pattern of phase-related feature selection, with some difference in few features, was also observable for other classification algorithms, which in contrasted resulted to have poorer performances. As noticeable from Fig. 6f, on average, there was no feature selected significantly more often than others, apart from the fruit size feature, which was decided to be always included in the first feature importance evaluation. This outcome, which might result from a random feature selection of the poorly performing models, supported the decision to first manually select the most important feature to use in the algorithms training and the quality of the overall method presented in Fig. 2.

Despite having good results, a possible approach to further enhance the impact of feature reduction on the classification performance, to be carried out in future works, would ideally follow two opposite directions. First, the use of the same automatic procedure starting from a bigger fraction of the dataset (higher than 18 features) and second, to directly use deep learning algorithms, which will include the feature selection procedure in the training of the discrimination algorithms. While the latter will most likely provide better results in terms of performance, the former would be useful to further understand the impact of each of the bioimpedance parameters on the classification of fruit ripening stage.

### Algorithm optimization results

**Figure 7:**
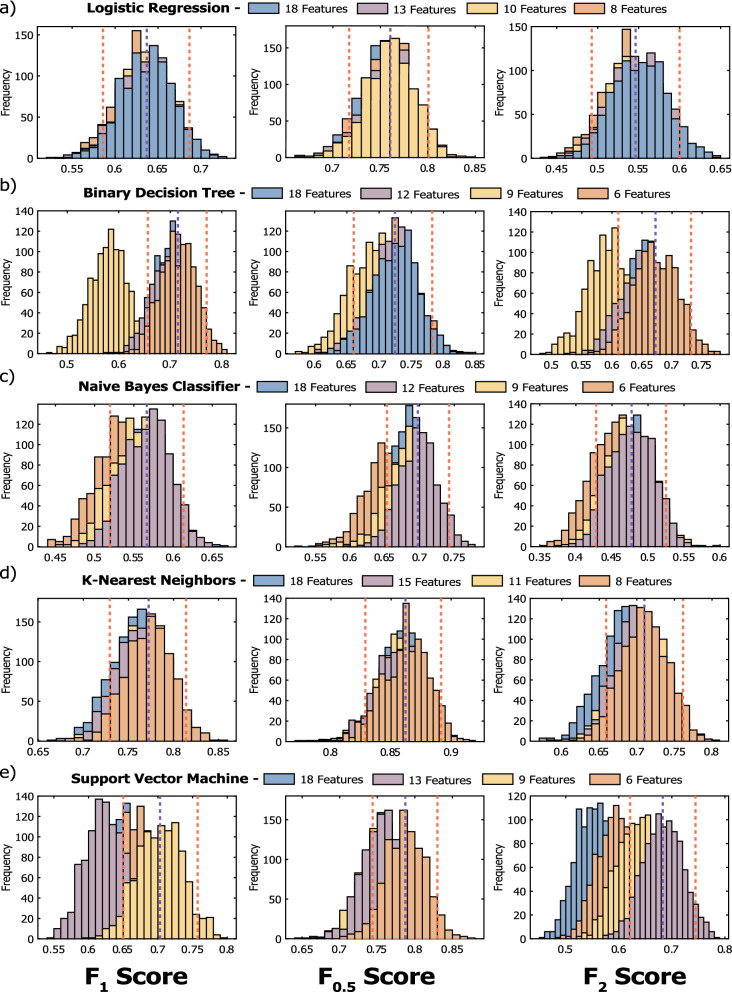
Bootstrapping validation F$_\beta$-scores for $\beta =$ 1, 0.5 and 2 for 10,000 round of validation for LR (**a**), DT (**b**), NBC (**c**), KNN (**d**) and SVM (**e**). Each number of feature validation performance is presented, together with the median F$_\beta$-score (red dashed line) and lower and upper confidence interval (95%, blue dashed line) for the best model.

The results of the bootstrap validation are presented in Supplementary Table 1 and Fig. 7, in terms of average F$_1$-score, F$_{0.5}$-score and F$_2$-score, together with the selected hyperparameters. The bootstrapping validation results were useful to evaluate the change in performance of the models when changing the number of the starting 18 features, which affected differently each considered classification algorithm. As regards to *L* *R* (Fig. 7a), there was no clear change in the model performance with the change of training feature number, probably due to the fact that it already achieved its maximum potential with all the 18 features. The same behavior was also observed for *D* *T*, where only the models developed with 9 features appears to have worse validation performances, probably due to different selected hyperparameters. As regards to *NBC*, the worst performing model, it appears that it had benefits from a mild feature reduction, from 18 to 12 features, only in the case of the F$_1$-score selection, while a further reduction had a negative effect on the model performance. Conversely, *KNN* appears to be the classification algorithm benefiting the most from a strong feature reduction, achieving the best validation results with 8 features, the lowest feature number. Finally, in the case of *SVM*, there was always a performance improvement with the feature reduction, which was different for each F$_\beta$-score selection. In fact, the model optimized toward the maximization of the F$_1$-score resulted to be better with 9 features, while the ones optimized towards the F$_{0.5}$ and F$_2$ scores, resulted to be better performing using 6 and 13 features, respectively.

### Classification results

Table 2 presents the results of the strawberry quality classification performed with each of the best optimized models, in terms of training and external test F$_1$, F$_{0.5}$ and F$_2$ scores, together with the number of features used in the model. Regarding the models developed towards the maximization of the F$_1$-score, the best training results were achieved by *KNN* using 8 features, with a F$_1$-score of 0.772, which in the test with the external dataset was reduced to 0.699 (− 9.5 %), showing a good stability of the algorithm in the prediction of unseen data. However, despite the lower training F$_1$-score, both *MLP* networks improved the test performance, achieving the best test F$_1$-score of 0.722 (+ 20.3 %) and 0.709 (+ 15.8 %) for the networks with 1 and 2 layers of neurons, respectively. The same performance pattern was also observed for both the models optimized for F$_{0.5}$ and F$_2$-scores, where the best training performance was achieved by *KNN* and the best test performance was obtained using *MLPs*, with a F$_{0.5}$-score of 0.817 and a F$_2$-score of 0.73 for the 2-layer MLP network. In general, except for the MLPs, it is possible to notice how the test F$_\beta$ scores are consistently lower than the training ones, with a 60.7% maximum reduction of the performance in the case of the 6-features *SVM* network optimized for the F$_{0.5}$-score. This emphasizes a lack of generalization of the models and most probably an overfitting problem on the training data, aspects that need to be taken into account in the evaluation of the possible use of such algorithms for the study case. On the other hand, *MLP* networks showed a consistent improvement in the testing performance, from a minimum of 15.8% to a maximum of 29%, showing good generalization capacity on unseen data. However, as it is commonly the opposite case, this aspect can also indicate a certain grade of instability of the network, which might have helped the obtainment of this result and might have been caused by 3 main factors. First, while in the training set the distribution of the 2 classes was skewed (5:1), in the test set classes distribution was balanced (1:1). This was partially mitigated in the models training considering the classes distribution as uniform, yet it is not to exclude that it might have had a different impact on different algorithms. Secondly, as it is also possible to notice from Fig. 4, there was a high degree of overlap between the two classes. This, as also observed by Schutten and Wiering in the case of SVM^48^, might have triggered a different classification rationale between the two sets, especially for points close to the decision boundary between the two classes, misclassified in the training and better classified in the differently distributed test set. Finally, this difference might have been caused by the presence of data points harder to classify in the training set with respect to the test set, with a consequent underfitting of the former. This aspect, which is not the main focus of this work, will be considered in future studies by tweaking the MLP hyperparameters such as the learning rate.

**Table 2:** External test results of the optimized models on the external unseen test dataset.

Algorithm	F$_1$ Score				F$_{0.5}$ Score				F$_2$ Score			
	Feat. Nr.	Train	Test	% Diff.	Feat. Nr.	Train	Test	% Diff.	Feat. Nr.	Train	Test F	% Diff.
LR	18	0.637	0.537	− 15.7	10	0.760	0.501	− 34.1	18	0.551	0.560	+ 1.6
DT	6	0.715	0.470	− 34.3	18	0.725	0.417	− 42.5	6	0.672	0.424	− 36.9
NBC	12	0.567	0.515	− 9.2	12	0.697	0.522	− 25.1	12	0.477	0.524	+ 9.9
KNN	8	0.772	0.699	− 9.5	8	0.862	0.665	− 22.9	8	0.710	0.687	− 3.2
SVM	9	0.703	0.508	− 27.7	6	0.787	0.309	− 60.7	13	0.683	0.581	− 14.9
MLP–1	18	0.600	0.722	+ 20.3	18	0.651	0.758	+ 16.3	18	0.547	0.649	+ 18.6
MLP–2	18	0.612	0.709	+ 15.8	18	0.694	0.817	+ 17.7	18	0.566	0.730	+ 29.0

For each model it is indicated the number of features, together with the average training and test F$_1$, F$_{0.5}$ and F$_2$ scores, and the difference between them.

Nevertheless, when training a machine learning algorithm, the training accuracy has a minor impact in the evaluation of the model performance compared to the test accuracy. Hence, considering the final test F$_\beta$ scores and the differences in this metric between the training and the test, MLP networks appear to be the most suitable candidate for an application in the on-field discrimination of strawberry ripening stage. It is important to highlight that MLPs achieved these results despite being developed using a smaller training set (due to the 60:20:20 training, validation, and internal testing split) and with a basic optimization of the network architecture. In this case, the 60:20:20 split combined with the internal testing for early stopping might have helped in obtaining a better generalizing model, despite being trained using fewer observations, thus avoiding overfitting. On the other hand, the other methods used a tenfold CV, which averages measures of fitness in prediction to derive a more accurate estimate of model prediction performance which, as observed in this case, might have produced models performing well on training and poorly on unseen data. In fact, except for *KNN*, that showed both good performance and stability, the other considered methods (i.e. *LR, DT, NBC and SVM*) resulted to not be ideal candidates for the use in the classification of strawberries from bioimpedance data, due to both a lack of generalization capability and poor performances. In particular, *SVM* poor performance resulted to be particularly surprising, as such method is commonly employed to carry out complex tasks, such as the one presented in this work. In this specific case, this was most likely caused by the above-mentioned class overlapping, triggering a different classification rationale between the two training and test sets, especially for points close to the decision boundary between the two classes. These problems, associated with the unsuitability of the above-mentioned algorithms for this specific classification, might have been enhanced by other factors, such as the training with severely skewed data and the bioimpedance data collection procedure, carried out with different electrode placement for each fruit.

Compared to previously mentioned works, presenting a 90% precision in the determination of avocado post-harvest ripening degree^28^ using ANN and 100% in the discrimination of frozen lemons using SVM^29^, the on-plant discrimination of strawberry ripening degree (0.72 F$_1$-score, MLP-1) resulted to be less effective. On the other hand, such works were carried out with a smaller number of fruit samples (respectively 100 and 180, compared to the 923 used in this work) and do not indicate if the test set was left unseen until the final classification task or was used in the training. Such aspects, while reinforcing the need to have a clear data analysis pipeline, might also practically lead to a high degree of overfitting in the developed models, leading to poor classification performances when employed in an on-site application.

Regarding the development of strawberry ripening classification models, and considering the above-mentioned issues, future work would ideally regard the standardization of the collection procedure with a fixed electrode distance for each fruit. This will allow the exclusion of the fruit size from the features used in the classification, leaving just bioimpedance-derived indexes, and the enhancement of the weight in the classification of features, such as the impedance magnitude, which are more affected by the electrode positioning. Furthermore, the Keysight e4990A used to acquire the bioimpedance data is considered to be a source of possible residual effects in the measurement, especially at high and low frequencies^49^. For this reason, future work will be focused on both the compensation of such issue and on the testing of bioimpedance data acquired from more reliable instrumentation to further validate the accuracy of our discrimination model. Additionally, aside from the use of better collected bioimpedance data, all the algorithms would benefit from the use of a larger dataset, which would both mitigate the effect of the class imbalance and help improving the models generalization performances. Finally, the data analysis pipeline presented in this paper will be applied to the development of discrimination models from bioimpedance data acquired from an already developed AD5933-based impedance analyzer^30^. Such models will be then integrated in the portable system for a live and on-site fruit quality discrimination, which could be either performed directly on-chip^50^ or on-cloud^51^, exploiting the system Bluetooth for data communication and result visualization, directly on a smartphone.

The implementation of the developed classification method during the fruits delivery by the farmers to the wholesaler (e.g. a cooperative for the distribution of the fresh product to the market) and/or the food processing industry could allow a fast, accurate and non-destructive classification of the product, yielding a more appropriate evaluation of its market value on the basis of its quality. Furthermore, this device and this application are even more interesting for those fruits (e.g. raspberries) for which the exact moment of the technological maturity is not yet fully identified.

## Conclusions

In this work, 6 of the most commonly used supervised machine learning classification models (i.e. LR, DT, NBC, KNN, SVM and MLP) were developed, optimized, tested, and compared for what concerns their performance in the discrimination of the ripening of strawberries from on-plant bioimpedance data, acquired in the 20 Hz–300 kHz frequency range. Furthermore, such classification methods were optimized developing a complete feature selection and optimization pipeline, not yet developed for bioimpedance data from fruit, paving the way for the optimization of future classification models development for the evaluation of fruit quality. The classification results, which benefited from the inclusion of the fruit size as an additional feature to the bioimpedance derived-ones, highlighted that two algorithms, i.e. KNN and MLP, were more effective in the discrimination of strawberry ripening stage. The first one showed good F$_\beta$-score stability from the training to the testing, especially for a balanced classification optimized towards the maximization of the F$_1$-score, with a 9.5% performance reduction. On the other hand, the latter, despite having worse training F$_\beta$-scores, showed better results when applied on the unseen test dataset, obtaining the highest F$_{0.5}$-score (0.817). This good accuracy, make MLP networks the best candidate for an on-field application of this technique for strawberry ripening monitoring. Such models, if properly integrated with portable impedance analyzers and non-destructive contact electrodes, shows to have a great potential for a real-time and on-field fruit quality assessment and ripening analysis. Such handheld integration, coupled with the rapidity and easiness of use of the bioimpedance technique, could greatly improve the harvesting efficiency, both in terms of saved time and quality of the harvested product, improving the profit margin of the strawberry producers.

## Supplementary Information

Supplementary Information.
