# Supplementary material for: Supervised binary classification methods for strawberry ripeness discrimination from bioimpedance data
Source: Sci Rep. 2021 May 27;11:11202. doi: 10.1038/s41598-021-90471-5 (PMC8160339; doi:10.1038/s41598-021-90471-5)
Supplement: Supplementary file 1 — Supplementary Information. [file 41598_2021_90471_MOESM1_ESM.pdf]

Appendix I

Bootstrap F-Score Validation and Selected Hyperparameters

LR	Selection For:	Feature Nr.	Mean F1	Mean F05	Mean F2	H1 - Lambda	H2 - Regularization	H3	H4
	**F1 Score**	18	0.637	0.755	0.551	2.11E-05	'ridge'	–	–
		8	0.630	0.759	0.539	2.11E-05	'ridge'	–	–
		10	0.636	0.760	0.546	1.87E–06	'ridge'	–	–
		13	0.636	0.757	0.548	6.29E–06	'ridge'	–	–
	**F0.5 Score**	18	0.633	0.754	0.546	7.10E–05	'ridge'	–	–
		8	0.630	0.759	0.539	2.11E–05	'ridge'	–	–
		10	0.636	0.760	0.546	1.87E–06	'ridge'	–	–
		13	0.636	0.757	0.548	6.29E–06	'ridge'	–	–
	**F2 Score**	18	0.637	0.755	0.551	2.11E–05	'ridge'	–	–
		8	0.630	0.759	0.539	2.11E–05	'ridge'	–	–
		10	0.636	0.760	0.546	1.87E–06	'ridge'	–	–
		13	0.636	0.757	0.548	6.29E–06	'ridge'	–	–
**DT**	**Selection For:**	**Feature Nr.**	**Mean F1**	**Mean F05**	**Mean F2**	**H1 – Min Leaf Size**	**H2 – Max Splits Nr..**	**H3 – Split Criterion**	**H4**
	**F1 Score**	18	0.6988	0.7540	0.6515	4	680	'deviance'	–
		6	0.7149	0.7634	0.6724	2	680	'deviance'	–
		9	0.5801	0.6870	0.5025	25	37	'deviance'	–
		12	0.7020	0.7564	0.6553	2	37	'deviance'	–
	**F0.5 Score**	18	0.6131	0.7246	0.5320	1	18	'deviance'	–
		6	0.6031	0.7215	0.5185	7	18	'deviance'	–
		9	0.5801	0.6870	0.5025	25	37	'deviance'	–
		12	0.6069	0.7208	0.5246	7	18	'deviance'	–
	**F2 Score**	18	0.6988	0.7540	0.6515	4	680	'deviance'	–
		6	0.7149	0.7634	0.6724	2	680	'deviance'	–
		9	0.6541	0.7299	0.5928	7	329	'gdi'	–
		12	0.7020	0.7564	0.6553	2	37	'deviance'	–
**NBC**	**Selection For:**	**Feature Nr.**	**Mean F1**	**Mean F05**	**Mean F2**	**H1 – Distribution**	**H2 – Kernel**	**H3 – Width**	**H4**
	**F1 Score**	18	0.563	0.695	0.474	'kernel'	'normal'	0.1376	–
		6	0.533	0.653	0.451	'kernel'	'box'	0.3235	–
		9	0.553	0.664	0.473	'kernel'	'box'	0.1891	–
		12	0.567	0.697	0.477	'kernel'	'normal'	0.1891	–
	**F0.5 Score**	18	0.486	0.680	0.378	'kernel'	'normal'	1.2758	–
		6	0.533	0.653	0.451	'kernel'	'box'	0.3235	–
		9	0.517	0.678	0.420	'kernel'	'triangle'	10.0642	–
		12	0.567	0.697	0.477	'kernel'	'normal'	0.1891	–
	**F2 Score**	18	0.563	0.695	0.474	'kernel'	'normal'	0.1376	–
		6	0.533	0.653	0.451	'kernel'	'box'	0.3235	–
		9	0.553	0.664	0.473	'kernel'	'box'	0.1891	–
		12	0.567	0.697	0.477	'kernel'	'normal'	0.1891	–
**KNN**	**Selection For:**	**Feature Nr.**	**Mean F1**	**Mean F05**	**Mean F2**	**H1 – Nr. Neighbors**	**H2 – Distance**	**H3 – Distance Weight**	**H4**
	**F1 Score**	18	0.754	0.847	0.681	93	'euclidean'	'inverse'	–
		8	0.772	0.847	0.710	13	'mahalanobis'	'squaredinverse'	–
		11	0.769	0.850	0.703	49	'spearman'	'inverse'	–
		15	0.762	0.839	0.699	26	'minkowski'	'squaredinverse'	–
	**F0.5 Score**	18	0.737	0.858	0.647	93	'mahalanobis'	'squaredinverse'	–
		8	0.764	0.862	0.687	93	'mahalanobis'	'squaredinverse'	–
		11	0.765	0.857	0.691	341	'minkowski'	'squaredinverse'	–
		15	0.755	0.858	0.674	178	'cityblock'	'squaredinverse'	–
	**F2 Score**	18	0.755	0.847	0.681	93	'euclidean'	'inverse'	–
		8	0.772	0.846	0.710	13	'mahalanobis'	'squaredinverse'	–
		11	0.770	0.851	0.703	49	'spearman'	'inverse'	–
		15	0.760	0.832	0.701	13	'seuclidean'	'squaredinverse'	–
**SVM**	**Selection For:**	**Feature Nr.**	**Mean F1**	**Mean F05**	**Mean F2**	**H1 – Kernel Scale**	**H2 – Box Constraint**	**H3 – Kernel Function**	**H4 – Polynomial Order**
	**F1 Score**	18	0.640	0.756	0.555	1	215.443	'linear'	–
		6	0.671	0.788	0.585	1	215.443	'polynomial'	2
		9	0.703	0.792	0.632	1	215.443	'polynomial'	2
		13	0.624	0.754	0.533	1	2.154	'linear'	–
	**F0.5 Score**	18	0.640	0.755	0.555	1	215.443	'linear'	–
		6	0.671	0.787	0.585	1	215.443	'polynomial'	2
		9	0.623	0.753	0.532	1	2.154	'linear'	–
		13	0.624	0.754	0.533	1	2.154	'linear'	–
	**F2 Score**	18	0.638	0.756	0.552	1	46.416	'linear'	–
		6	0.671	0.768	0.596	1	1000	'polynomial'	2
		9	0.703	0.792	0.632	1	215.443	'polynomial'	2
		13	0.739	0.805	0.683	1	46.416	'polynomial'	2

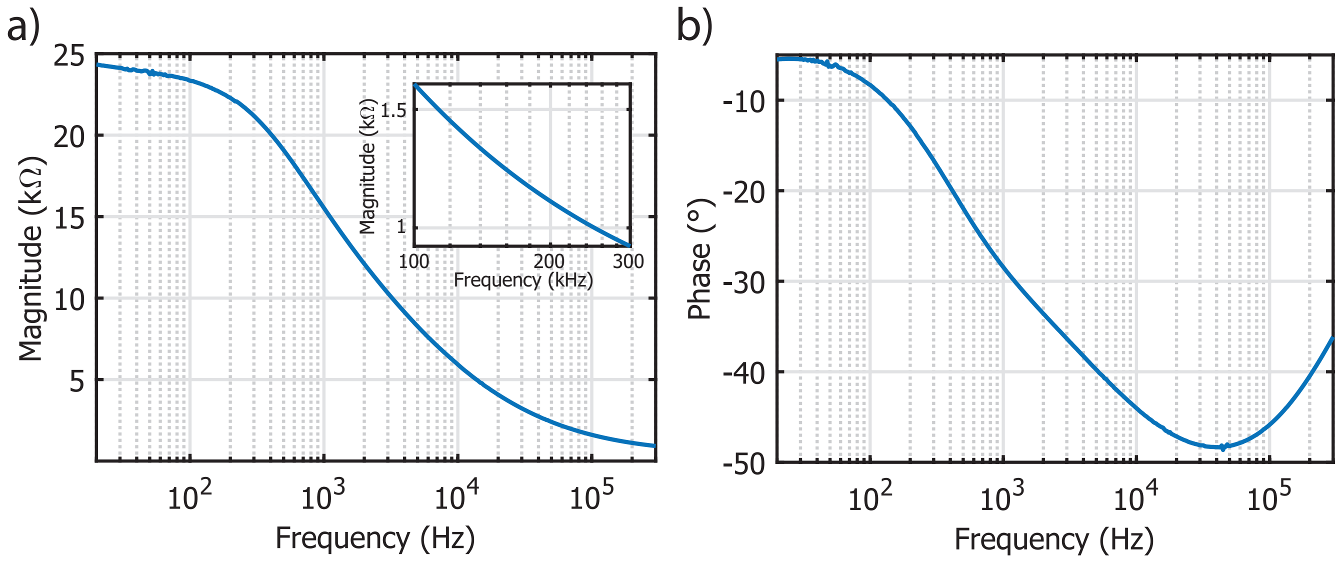

Figure 1. Single fruit magnitude (a) and phase (b) measurement example. Inset graph in (a) represents a magnification of the 100–300 kHz frequency points.
